# Plastoquinone Lipids: Their Synthesis via a Bifunctional Gene and Physiological Function in a Euryhaline Cyanobacterium, *Synechococcus* sp. PCC 7002

**DOI:** 10.3390/microorganisms11051177

**Published:** 2023-04-30

**Authors:** Mimari Kondo, Motohide Aoki, Kazuho Hirai, Ryo Ito, Mikio Tsuzuki, Norihiro Sato

**Affiliations:** School of Life Sciences, Tokyo University of Pharmacy and Life Sciences, Hachioji, Tokyo 192-0392, Japan

**Keywords:** acyl plastoquinol, NaCl-stress acclimatization, plastoquinone-B, plastoquinone-C acyltransferase, sedimented-cell growth, static culture, *Synechococcus* sp. PCC 7002

## Abstract

Eukaryotic photosynthetic organisms synthesize triacylglycerols, which are crucial physiologically as major carbon and energy storage compounds and commercially as food oils and raw materials for carbon-neutral biofuel production. TLC analysis has revealed triacylglycerols are present in several cyanobacteria. However, mass spectrometric analysis has shown that freshwater cyanobacterium, *Synechocystis* sp. PCC 6803, contains plastoquinone-B and acyl plastoquinol with triacylglycerol-like TLC mobility, concomitantly with the absence of triacylglycerol. *Synechocystis* contains *slr2103*, which is responsible for the bifunctional synthesis of plastoquinone-B and acyl plastoquinol and also for NaCl-stress acclimatizing cell growth. However, information is limited on the taxonomical distribution of these plastoquinone lipids, and their synthesis genes and physiological roles in cyanobacteria. In this study, a euryhaline cyanobacterium, *Synechococcus* sp. PCC 7002, shows the same plastoquinone lipids as those in *Synechocystis*, although the levels are much lower than in *Synechocystis*, triacylglycerol being absent. Furthermore, through an analysis of a disruptant to the homolog of *slr2103* in *Synechococcus*, it is found that the *slr2103* homolog in *Synechococcus*, similar to *slr2103* in *Synechocystis*, contributes bifunctionally to the synthesis of plastoquinone-B and acyl plastoquinol; however, the extent of the contribution of the homolog gene to NaCl acclimatization is smaller than that of *slr2103* in *Synechocystis*. These observations suggest strain- or ecoregion-dependent development of the physiological roles of plastoquinone lipids in cyanobacteria and show the necessity to re-evaluate previously identified cyanobacterial triacylglycerol through TLC analysis with mass spectrometric techniques.

## 1. Introduction

Plastoquinone (PQ)-related acylated lipids (herein referred to as PQ lipids), which are generally minor lipid components, have long been observed in seed plants [1], and they have also been reported in a freshwater cyanobacterium, *Synechocystis* sp. PCC 6803 (herein referred to as *Synechococystis* 6803) [2,3]. Recently, we classified PQ lipids in *Synechococystis* 6803 into two subclasses: (1) acyl hydroxy-plastoquinone (PQ-C), which is the same as the sole PQ lipid, PQ-B, in seed plants and (2) a novel PQ lipid, acyl plastoquinol (PQH_2_) [2,3]. In line with the discovery of PQ lipids in *Synechococystis* 6803, *slr2103*, a homolog of type-2 diacylglycerol acyltransferase genes, has been identified as the gene responsible for the synthesis of PQ-B and acyl plastoquinol, and it has been proposed to represent a bifunctional acyltransferase that uses plasoquinone-C and plastoquinol as acyl acceptor alchohols [3].

The study of seed plant PQ-B from physiological aspects has previously demonstrated its potential to function as an electron acceptor of PSII in tobacco thylakoid membranes and its quantitative increase in *A. thaliana* plants with aging during growth under high light conditions [4,5]. However, no conclusion has been drawn concerning the physiological roles of PQ-B in seed plants, PQ-B deficient mutants of which are unavailable, with no genes identified for PQ-B synthesis. Meanwhile, PQ lipids or *slr2103* have been shown to be crucial in *Synechococystis* 6803 for the growth of cells as a sediment in a static culture [3]. It is of particular note that PQ lipids or *slr2103* have been shown to facilitate the formation of a pellicle biofilm in *Synechococystis* 6803 cells under NaCl-stress conditions, thereby, allowing them to rapidly grow, owing to the high availability of CO_2_ and light energy for photosynthesis [3]. In this context, it is of interest that homologs of *slr2103* are present in numerous cyanobacteria collected from a wide variety of habitats that would least encounter fluctuations in saline stress, however, with the exclusion of oceanic *Synechococcus* and *Prochlorococcus* strains [3]. In addition, PQ lipid or *slr2103*-dependent formation of a pellicle biofilm has increased the potential to industrially utilize the gene for concentration of high-added-value biomass in photosynthetic microorganisms [3]. Moreover, notably, *slr2103* homologs are present in cyanobacterial species that naturally bloom such as *Microcystis* [3]. Therefore, elucidation of the molecular function of PQ lipids in the pellicle biofilm formation in *Synechocystis* would lead to a better understanding of the molecular mechanism of cyanobacterial natural blooms and accordingly would provide clues to control it [3].

Triacylglycerols (TGs) represent neutral lipids that function as carbon and energy storage compounds. Eukaryotic microorganisms that perform photosynthesis, or microalgae, accumulates TGs upon exposure to aberrant environmental conditions [6]. TG synthesis demands substantial amounts of chemical energy for fatty acid synthesis. The induction of TG accumulation in microalgae, therefore, is regarded to be a strategy to consume excess energy, owing to general repression in metabolism, and thereby, to repression of the generation of reactive oxygen species for cell survival [7]. In addition to the above-mentioned physiological aspects, much attention has been focused on algal TG in the biofuel production industry in view of the carbon neutrality of algal TG-based fuels [7]. Through TLC analyses, several cyanobacteria, including *Synechococystis* 6803, have been found to possess TG as a minor lipid [8,9,10,11,12,13,14]. However, recently, it has been reported that the above-mentioned PQ lipids in *Synechocystis* 6803 showed TG-like TLC mobility, with the absence of TG or its presence at a background level [3]. In view of the previous experimental limitation that TG and PQ lipids could not be distinguished through TLC analyses with the use of a conventional solvent system, the previous reports of other cyanobacterial TGs should, therefore, be re-evaluated, [3]. Intriguingly, *Nostoc punctiforme* and *Arthrospira platensis* (formally known as *Spirulina platensis*), which were previously reported to show TG on TLC analysis, possess *slr2103* homologs [3,9,14]. In this context, it is possible that cyanobacteria, which possess homologs of *slr2103*, synthesize PQ lipids but not TG [3].

A euryhaline cyanobacterium, *Synechococcus* sp. PCC 7002 (herein referred to as *Synechococcus* 7002), which is capable of natural transformation, contains *SYNPCC7002_A0918*, a homolog of *slr2103* (https://genome.microbedb.jp/cyanobase/GCA_000019485.1 accessed on 1 March 2023, 65.6% identity in the amino acid sequence) [3,15]. In this study, we examined the chemical structures of neutral lipids and the function of the *slr2103* homolog in *Synechococcus* 7002. In addition, we investigated whether or not the homolog gene or PQ lipids are responsible for cellular acclimatization to NaCl stress in *Synechococcus* 7002. The results obtained are discussed in the context of the evolutionary diversification of cyanobacteria.

## 2. Materials and Methods

### 2.1. Cyanobacterial Strains, Media, and Growth Conditions

The cyanobacterial strains used were *Synechococcus* 7002 (the Pasteur Culture Collection) and *Synechocystis* 6803 (glucose-tolerant strain [16]) and a mutant of *Synechococccus* 7002 (see below). The cells were cultured in BG-11 with illumination (50 μmol photons m^−2^ s^−1^) at 30 °C in a glass tube (50 mL, 3 cm in diameter) with bubble aeration of ordinary air (160 mL·min^−1^) or statically in a titer plate (1 mL in a well, 1 cm in diameter) [3]. Vitamin B12 was included in a *Synechococcus* 7002 culture (4 µg·mL^−1^). Cell growth was monitored by measuring the OD_730_ value or chlorophyll (Chl) content in the cultures with a spectrophotometer DU 640 (Beckman). Chl was extracted from the cells with 100% methanol, as described previously [3].

### 2.2. Preparation of Lipids and Analysis of Their Constituent Fatty Acids 

Total lipids were extracted from *Synechococcus* 7002 cells, according to a method for green algal cells, *Chlorella kessleri* [17], which is based on the method by Bligh and Dyer [18]. Briefly, cells equivalent to ca. 800–900 µg Chl were harvested by centrifugation, and then were subjected to lipid extraction with the use of methanol and chloroform. The extracted lipid solution was emulsified with the addition of distilled water. The resultant solution was centrifugated for separation into upper and lower layers, the latter of which was recovered as total lipids. Then, total lipids were separated as necessary into individual lipid classes by TLC [3]. TLC was performed with a solvent system of hexane/diethyl ether/acetate (70:30:1, by vol.) or 100% toluene for isolation of PQ lipids. Total lipids were also subjected for LC-MS and LC-MS^2^ analysis (see below).

### 2.3. MS Spectrometric Analysis

The lipid profiling of samples was performed with an LC-QqQ(LIT)-MS/MS system composed of a LC-20A Prominence series HPLC (Shimadzu, Kyoto, Japan), and a 3200 QTRAP hybrid triple quadrupole/linear ion trap mass spectrometer with Turbo V™ ion source (Sciex, Concord, ON, Canada), as previously described [3]. Enhanced mass scan (EMS) was used for the lipid signal survey with the positive electrospray ionization (ESI+) mode. The tandem MS (MS^2^) analysis of PQ lipid molecular species was carried out under the mode of EMS with independent data acquisition and enhanced product ion scans (EMS-IDA-EPI). Meanwhile, for high-resolution mass measurement, the LC-Q/TOF MS analysis was performed with a 1290 infinity II high-performance liquid chromatograph and a 6530 Accurate-Mass Q-TOF mass spectrometer equipped with a JetStream source (both, Agilent Technologies, Santa Clara, CA) in ESI+ mode [3]. A lock mass solution including purine (*m*/*z* 121.0509) and HP-921 (hexakis (1H,1H,3H-tetrafluoro-pentoxy) phosphazene) (*m*/*z* 922.0098) was utilized for the real-time lock mass correction. For chromatographic separation of total lipids, 2 µL of sample was injected into a CERI L-column2 ODS (100 × 2.1 mm, 3 µm, Tokyo, Japan), held at 40 °C. Mobile phase solvents and gradient conditions were as previously described [3].

### 2.4. Gene Manipulation in Synechococcus 7002

An ORF, *SYNPCC7002-A0918*, which is a homolog of *slr2103*, was disrupted in *Synechococcus* 7002, as previously described (https://genome.microbedb.jp/cyanobase/GCA_000019485.1/genes/SYNPCC7002_A0918, accessed on 12 April 2023) [3,15]. A genomic DNA fragment of 1.8 kbp, including the ORF, was amplified by PCR with primer set 1 (forward, 5′ AAACATGGGGTGGTTTGTCGG 3′ and reverse, 5′ GGTCATTAATGCGGCGATCG 3′). PCR was performed with Ex-Taq DNA polymerase (Takara) with the use of a T100 Thermal Cycler (BIO-RAD). The thermocycle program was as follows: 2 min at 95 °C, followed by 28 cycles of 10 s at 95 °C, 30 s at 60 °C, and 2 min at 72 °C, and finally by extension at 72 °C for 7 min. The PCR product was ligated to the pGEM T-EASY vector (Promega), cut with both BamHI and HindIII for 0.8 kbp deletion in the ORF, and then blunt-ended. The linear DNA obtained was ligated with the kanamycin-resistant gene in pKMT903 (constructed from the transposon Tn903 [19], a kind gift from Dr. K. Okada, Tokyo University of Pharmacy and Life Sciences) for generation of a plasmid to disrupt the ORF, which was then used for natural transformation in *Synechococcus* 7002 [15]. Briefly, *Synechococcus* 7002 cells were grown to OD_730_ of ca. 0.5, and were concentrated by 10-fold in a fresh medium through centrifugation. The concentrated culture (1 mL) was mixed with the plasmid DNA (3 μg) for further culturing for 12 h in the dark, and then was used for the selection of kanamycin-resistant transformants on an agar plate of BG-11 containing kanamycin (20 μg·mL^−1^). The complete disruption of the ORF was confirmed by genomic DNA PCR with primer set 2 (forward, 5′ ATGCCCCTTTTTCCGCCATT 3′ and reverse, 5′ CTAGGGACAACTTTTAGGGC 3′). PCR was performed, as described above, with the modification that 2 min at 72 °C was shortened to 1 min.

## 3. Results

### 3.1. The Presence of PQ Lipids in Synechococcus 7002

**Figure 1:**
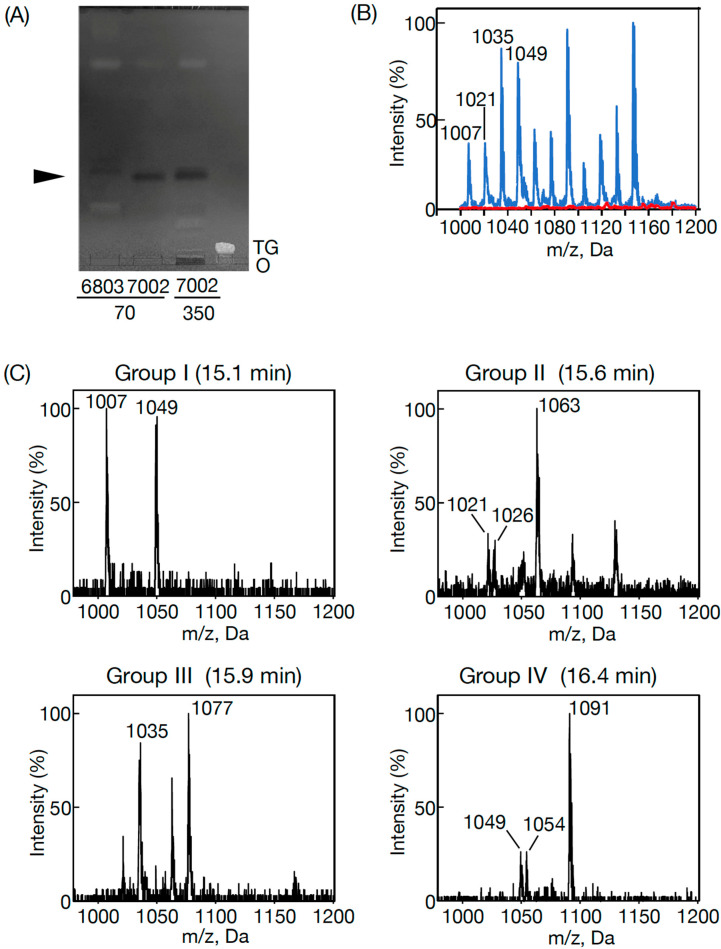
PQ lipids in *Synechococcus* 7002: (**A**) TLC profile of PQ lipids in *Synechococcus* 7002 (7002) and *Synechocystis* 6803 (6803) with the solvent system of 100% toluene. Total lipids extracted from *Synechococcus* 7002 cells equivalent to 70 or 350 OD_730_·mL culture (containing 602 and 3008 µg Chl, respectively) or those from *Synechocystis* 6803 cells equivalent to 70 OD_730_·mL culture (containing 434 µg Chl) were used to separate PQ lipids by TLC with the solvent system of hexane/diethyl ether/acetate (70:30:1 by vol.). PQ lipids were then subjected to another TLC with the solvent system of 100% toluene. The arrowhead indicates the position of PQ lipids. O, origin. TG indicates a marker TG. Note that PQ lipids are detectable in *Synechocystis* 6803 cells equivalent to 70 OD_730_·mL culture but not in *Synechococcus* 7002 cells, and that PQ lipids are still undetectable in *Synechococcus* 7002 cells equivalent to 350 OD_730_·mL culture. (**B**) The MS spectrum of a lipid fraction (retention time of 15–17 min, *m*/*z* 1000–1160), including PQ lipids, on LC-MS analysis of total lipids prepared from WT cells (blue) or Δ*slr2103* cells (red). The signal intensity of *m*/*z* 1091 relative to the total lipid fraction (retention time of 2–18 min, *m*/*z* 300–1200) in the WT was adjusted to 100%. (**C**) Classification of lipid molecules into four groups, Groups I-–V, by retention time on LC-MS analysis. Characteristic members were *m*/*z* 1007 and *m*/*z* 1049 in Group I (15.1 min); *m*/*z* 1021, *m*/*z* 1026, and *m*/*z* 1063 in Group II (15.6 min); *m*/*z* 1035 and *m*/*z* 1077 in Group III (15.8 min); and *m*/*z* 1049, *m*/*z* 1054 and *m*/*z* 1091 in Group IV (16.4 min). Note that this classification demonstrates that *m*/*z* 1007, *m*/*z* 1021, and *m*/*z* 1035 in (**B**) belong to Groups I–III, respectively (**C**), whereas *m*/*z* 1049 ion in (**B**) are composed of those belongjng to Groups I and IV (**C**).

The presence of a homolog of *slr2103* in *Synechococcus* 7002 raised the possibility that the cells contained PQ lipids [3]. However, the TLC analysis of lipids in *Synecococcus* 7002, distinct from those in *Synechocystis*, did not reveal a candidate for PQ lipids (Figure 1A). Meanwhile, the LC-MS analysis of total cellular lipids in *Synechococcus* 7002 definitely showed several signals in the region of *m*/*z* 1000–1160 at the retention time of 15–17 min, where PQ lipid ions were found in *Synechocystis* (Figure 1B) [3]. The lipid ions in *Synechococcus* 7002, similar to the PQ lipid ions in *Synechococystis* 6803, could be divided into retention time-dependent Groups I–IV, which included characteristic members: *m*/*z* 1007 and *m*/*z* 1049 in Group I (15.1 min); *m*/*z* 1021, *m*/*z* 1026, and *m*/*z* 1063 in Group II (15.6 min); *m*/*z* 1035 and *m*/*z* 1077 in Group III (15.8 min); and *m*/*z* 1049, *m*/*z* 1054, and *m*/*z* 1091 in Group IV (16.4 min) (Figure 1C). Thus, it was revealed that individual groups in *Synechococcus* 7002 possessed two or three members among four in the corresponding *Synechococystis* 6803 groups, at least judging from their *m*/*z* values [3].

**Figure 2:**
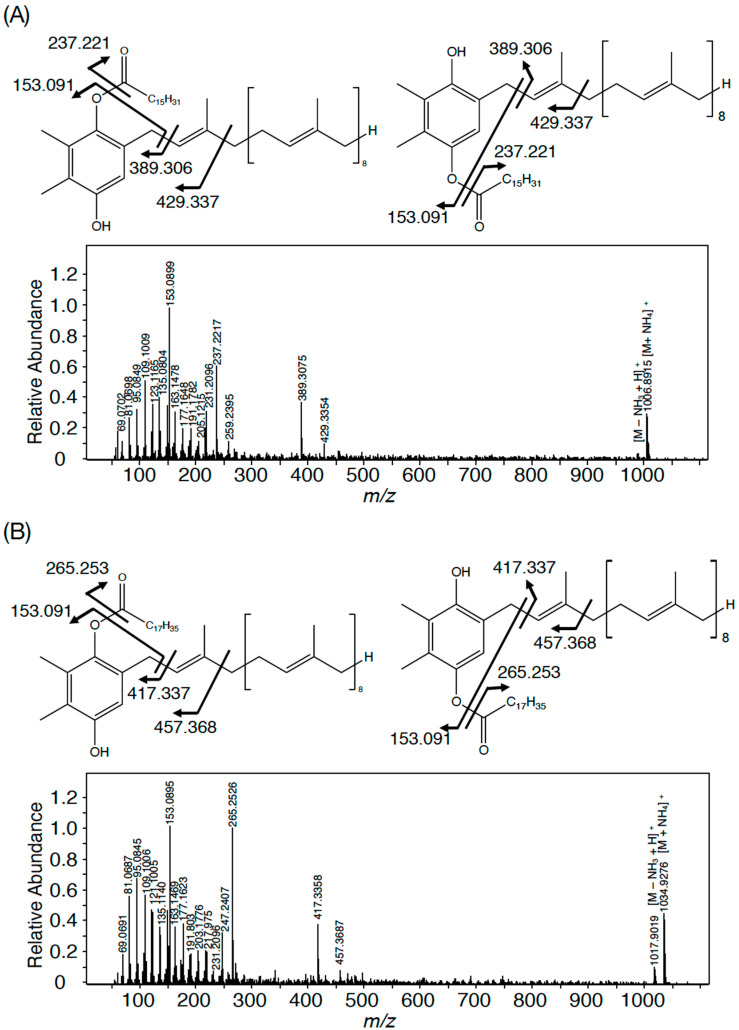
High-resolution MS^2^ spectra of *m*/*z* 1007 (**A**) and *m*/*z* 1035 (**B**) in *Synechococcus 7002*. Two possible structures of acyl plastoquinol are tentatively shown.

**Figure 3:**
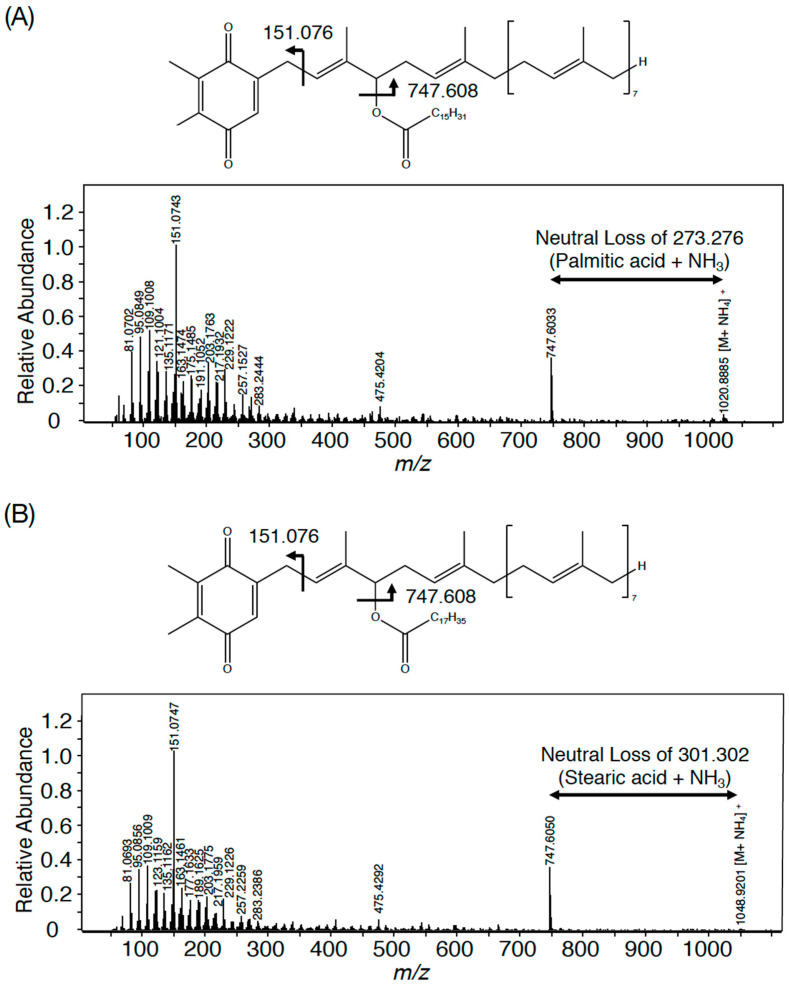
High-resolution MS^2^ spectra of *m*/*z* 1021 (**A**) and *m*/*z* 1049 (**B**) in *Synechococcus* 7002. The structure of acyl PQ-C is tentatively shown to possess the ester bond in the first isoprene unit.

In *Synechocystis* 6803, four members in each group represent the same PQ lipid molecular species with adduction of four distinct ions, for example, the member with the lowest *m*/*z* value in each group represents an NH_4_^+^-adducted lipid ion [3]. The ions in *Synechococcus* 7002, which corresponded to those with the lowest *m*/*z* values in *Synechocystis* 6803, i.e., *m*/*z* 1007 in Group I, *m*/*z* 1021 in Group II, *m*/*z* 1035 in Group III, and *m*/*z* 1049 in Group IV, were individually analyzed by high-resolution MS spectrometry to compare the MS spectra with those of their counterparts in *Synechocystis* 6803. As shown in Figure 2A, the accurate masses of *m*/*z* 1007 and *m*/*z* 1035 in *Synechococcus* 7002 were 1006.89 and 1034.93, which matched the counterparts of NH_4_^+^-adducted palmitoyl PQH_2_ (1006.90) and stearoyl PQH_2_ (1034.93), respectively [3]. Furthermore, the high-resolution MS^2^ spectra of *m*/*z* 1007 and *m*/*z* 1035 ions, respectively, showed fragment ions of spiked signals below at *m*/*z* 300 and four specific fragment ions, which were characteristic of NH_4_^+^-adducted palmitoyl PQH_2_ and staroyl PQH_2_ in *Synechocystis* 6803 (Figure 2 and Table 1) [3]. Meanwhile, *m*/*z* 1021 and *m*/*z* 1049 in *Synechococcus* 7002 indicated 1020.89 and 1048.92, respectively, which corresponded to NH_4_^+^-adducted palmitoyl PQ-C and stearoyl PQ-C in *Synechocystis* 6803 (Figure 3) [3]. Moreover, *m*/*z* 1021 and *m*/*z* 1049 were almost the same as NH_4_^+^-adducted palmitoyl PQ-C and stearoyl PQ-C, respectively, in fragment ion patterns, including *m*/*z* 747.6 that was generated through the neutral loss of 16:0 or 18:0, in addition to similar spiked signals to those of palmitoyl PQH_2_ and stearoyl PQH_2_ (Figure 2 and Figure 3 and Table 1). Collectively, it was shown that *m*/*z* 1007 and *m*/*z* 1035 in *Synechococcus* 7002 represented palmitoyl PQH_2_ and stearoyl PQH_2_, respectively, whereas *m*/*z* 1021 and *m*/*z* 1049 corresponded to palmitoyl PQ-C and stearoyl PQ-C, respectively. However, it should be emphasized that the PQ lipid content was at least five-fold lower in *Synecococcus* 7002 than in *Synechocystis* 6803 (Figure 1A). The loss of one or two members in Groups I–IV in *Synechococcus* 7002, relative to four in *Synechococystis* 6803, might reflect some difference in the ion species-dependent adduction efficiency of PQ lipids in total lipid samples between these two cyanobacteria (Figure 1B,C) [3].

The top twenty product ions at high signal intensities are shown in the respective standard PQ lipids, palmitoyl PQH_2_, stearoyl PQH_2_, palmitoyl PQ-C, stearoyl PQ-C, and PQ-C in *Synechocystis* 6803 for comparison with their counterparts in PQ lipids in *Synechococcus* 7002. Plus (+) and minus (−) indicate product ions present and absent in *Synechococcus* 7002 PQ lipids, within the top thirty at high signal intensities.

### 3.2. Essentiality of SYNPCC7002-A0918, an slr2103 Homolog, for PQ Lipid Synthesis in Synechococcus 7002

**Figure 4:**
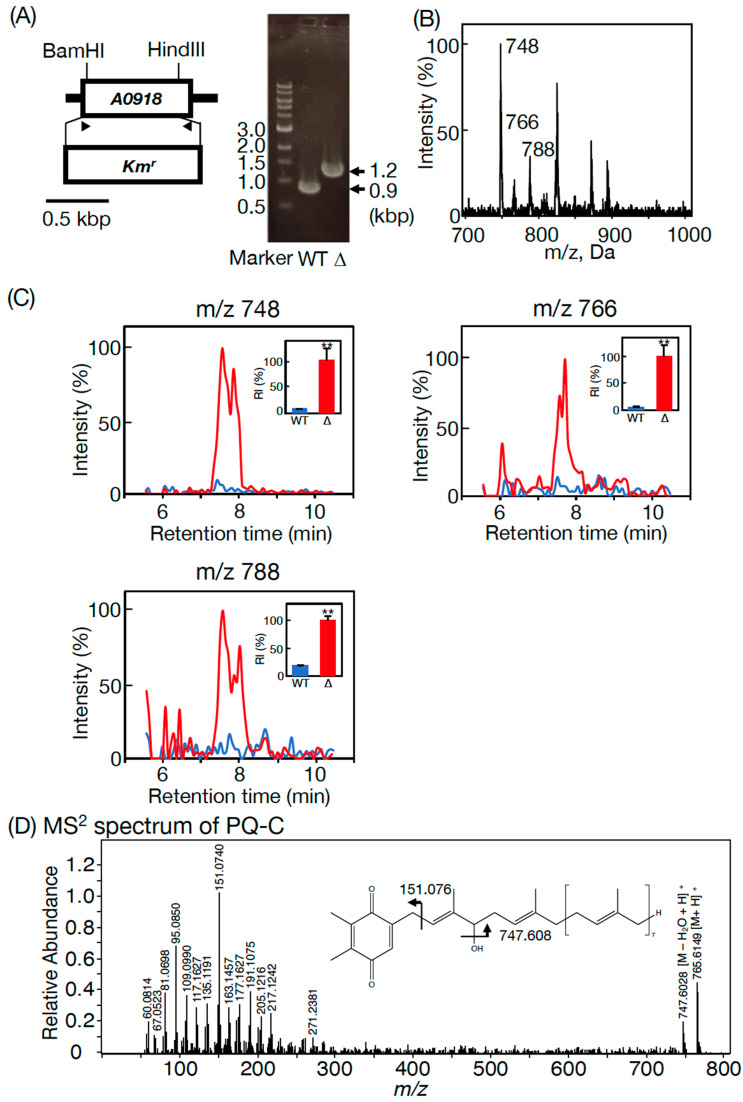
Lipid phenotypes of Δ*SYNPCC7002-A0918*: (**A**) Insertional mutagenesis strategy (left) and confirmation of *SYNPCC7002-A0918* disruption by PCR with genomic DNA as a template in *Synechococcus 7002* (right). Arrowheads indicate a primer set for the genomic PCR; (**B**) a prominent signal at *m*/*z* 748, followed by two weaker signals at *m*/*z* 766 and *m*/*z* 788 at 7.6 min on LC-MS analysis of total lipids in Δ*SYNPCC7002-A0918*; (**C**) Much stronger signal intensity in Δ*SYNPCC7002-A0918* (red) than in the WT (blue) at *m*/*z* 748, *m*/*z* 766, or *m*/*z* 788, as revealed on the LC-MS chromatogram of total lipids at 7.6–7.8 min. The signal intensity of the highest peak relative to a total lipid fraction (retention time of 2–18 min, *m*/*z* 300–1200) in the WT was adjusted to 100%; (**D**) the MS^2^ spectrum of *m*/*z* 766 in Δ*SYNPCC7002-A0918*, which was eluted at 7.6 min on the LC-MS analysis. The structure of PQ-C with the hydroxy group at the first isoprene unit is tentatively shown.

An ORF, *SYNPCC7002-A0918*, in *Synechococcus* 7002 is a homolog of *slr2103* in *Synechococystis* 6803. A previous RNA-seq analysis in *Synechococcus* 7002 demonstrated that *SYNPCC7002-A0918* was expressed at the transcript level [20]. To obtain insights into PQ lipid synthesis in *Synechococcus* 7002, its ORF, *SYNPCC7002-A0918*, a homolog of *slr2103* in *Synechococystis* 6803, was disrupted through homologous recombination (Figure 4A) [3]. The disruptant (Δ*SYNPCC7002-A0918*) was found to have lost the whole set of PQ lipid molecules, as revealed in the LC-MS spectrum of total cellular lipids (Figure 1B). We also observed *m*/*z* 748, *m*/*z* 766, and *m*/*z* 788 signals on the LC-MS spectrum of total lipids at 7.6 min in Δ*SYNPCC7002-A0918* (Figure 4B). As compared with Δ*SYNPCC7002-A0918*, however, the WT showed markedly lower intensities of the *m*/*z* 748, *m*/*z* 766, and *m*/*z* 788 signals at 7.6–8.0 min on the LC-MS chromatogram of total lipids (Figure 4C). The abnormally strong signals of these three ions in Δ*SYNPCC7002-A0918* were similarly reported in *Synechococystis* 6803 Δ*slr2103* when compared with its WT [3]. Notedly, the *m*/*z* 748, *m*/*z* 766, or *m*/*z* 788 signals were composed of multiple peaks (Figure 4C). The high-resolution LC-MS spectrum of *m*/*z* 766 at 7.6 min was almost the same as that of the standard PQ-C of *C. reinhardtii* (Figure 4D and Table 1) [3]. Similar to *Synechococystis* 6803, therefore, the *m*/*z* 748, 766, and 788 ions represent PQ-C with three distinct ionization patterns, i.e., [M-H_2_O+H]^+^, [M+H]^+^, and [M+Na]^+^, respectively, and that their multiple peak signals reflect the presence of PQ-C isomers with respect to the position of the isoprenoid unit to which a hydroxy group is bound (Figure 4C,D) [3]. From the loss of palmitoyl PQ-C and stearoyl PQ-C in Δ*SYNPCC7002-A0918*, concomitantly with accumulation of PQ-C, the postulated acyl-acceptor substrate of PQ-C acyltransferase, it can be deduced that *SYNPCC7002-A0918* is the gene for the PQ-C acyltransferase. Moreover, additional loss of acyl PQH_2_ in Δ*SYNPCC7002-A0918* indicates that *SYNPCC7002-A0918* also functions in the synthesis of palmitoyl PQH_2_ and stearoyl PQH_2_ (Figure 1B). 

### 3.3. Physiological Roles of PQ Lipids in Synechococcus 7002

**Figure 5:**
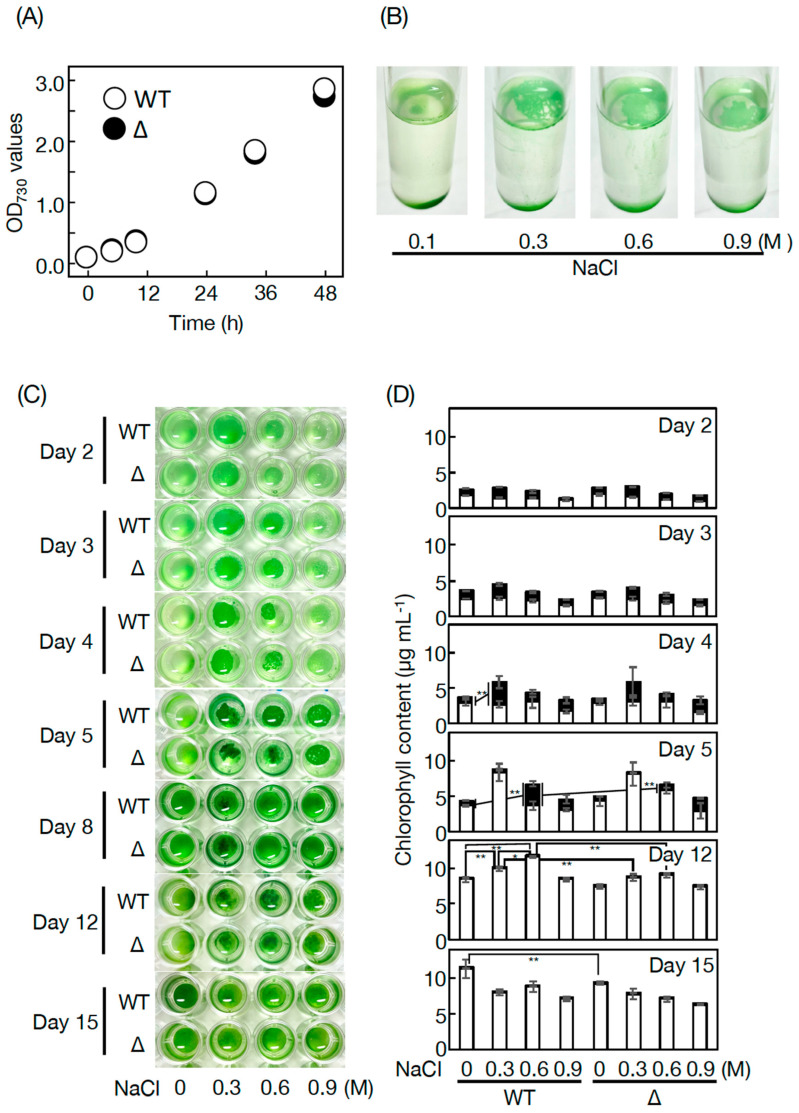
Cell growth of *Synechococcus* 7002 WT and Δ*SYNPCC7002-A0918*: (**A**) Cell growth in bubble aeration 50 mL cultures (WT, open circles and Δ*SYNPCC7002-A0918*, closed circles); (**B**) photographs of WT cells in a 1 mL static culture for 5 days with or without NaCl stress at from 0.3 to 0.9 M; (**C**) photographs of microtiter plates where the WT and Δ*SYNPCC7002-A0918* cells were statically cultured with or without NaCl stress at 0.3 to 0.9 M; (**D**) concomitant monitoring of cell growth based on the Chl content. The Chl content in the culture was adjusted to 0.86 ± 0.01 and 0.82 ± 0.02 µg·mL^−1^ in the WT and Δ*SYNPCC7002-A0918*, respectively, on Day 0. Open and closed bars correspond to non-floating and floating cells, respectively. The values shown are averages ± SD for three experiments. The significance of differences was evaluated by means of a Student’s *t*-test. * *p* < 0.1 and ** *p* < 0.05.

In place of the usually employed artificial seawater media such as medium A in *Synechococcus* 7002 [15], this study used a freshwater medium, BG-11, which allowed *Synechococcus* 7002 WT cells to grow vigorously, similar to *Synechocystis* 6803 cells (Figure 5A) [3]. Then, the physiological roles of PQ lipids were investigated through characterization of Δ*SYNPCC7002-A0918* (Figure 5). Cell growth was similar for the *Synechococcus* 7002 WT and Δ*SYNPCC7002-A0918* under bubble aeration conditions, which demonstrated no crucial roles of PQ lipids (Figure 5A). Then, we examined the responsibility of PQ lipids for stress acclimatization (Figure 5B–D). In a static culture where the CO_2_ and light energy supply would severely limit photosynthesis, as much as 17–36% of the cell population floated on the culture surface from Days 2 to 5 in *Synechococcus* 7002 WT (see 0 M NaCl). Since pellicle biofilm formation was observed similarly in Δ*SYNPCC7002-A0918*, with no growth defect relative to in the WT, it seemed that PQ lipids played no crucial roles in pellicle biofilm formation or cell growth at this early growth phase until Day 5 (Figure 5C,D). Further culturing from Days 12 to 15 demonstrated that WT and Δ*SYNPCC7002-A0918* cells both grew mainly as sediments. Intriguingly, Δ*SYNPCC7002-A0918* cells showed slightly but significantly delayed growth, relative to the WT cells, which inferred some role of PQ lipids in sedimented cell growth at this later phase under static conditions (Figure 5C,D, see Day 15).

Meanwhile, NaCl stress seemed to facilitate the formation of a pellicle biofilm in the WT *Synechococcus* 7002 (Figure 5B), in particular, on Days 4 and 5 at 0.3 M and 0.6 M NaCl, respectively (Figure 5B–D, compare corresponding black bars with their counterparts at 0 M NaCl). It was likely that, on Day 5, the statically cultured cells, owing to the high availability of CO_2_ and light energy for the floating cells, in total, achieved 1.8- and 1.5-fold higher growth levels at 0.3 and 0.6 M NaCl, respectively, relative to the non-NaCl-stressed cells. Later, cells proliferated, mainly as sediments at 0.3 and 0.6 M NaCl, to sustain their higher growth levels than at 0 M NaCl, until Day 12 (Figure 5C,D), with the pellicle biofilm sinking to the bottom by Day 8 (Figure 4C). Δ*SYNPCC7002-A0918* demonstrated WT-like pellicle-biofilm formation at 0.3 M but not at 0.6 M NaCl (Figure 5C,D, see Days 4 and 5). The failure to increase pellicle biofilm formation in Δ*SYNPCC7002-A0918* cells at 0.6 M NaCl seemed to adversely affect acclimatization of cell growth, in contrast to in the WT cells, which achieved the highest growth level at 0.6 M NaCl on Day 12 (Figure 5C,D, compare bars at 0.6 M NaCl on Day 12 between the WT and Δ*SYNPCC7002-A0918*). These results indicated some crucial role of PQ lipids in cellular acclimatization to severe NaCl stress through facilitation of pellicle biofilm formation. 

## 4. Discussion

In contrast to eukaryotic photosynthetic organisms that generally synthesize TG, only limited strains of cyanobacteria have thus far been reported to show TG. The results of this study revealed that bubble aeration cultured *Synechococcus* 7002 cells did not contain TG, as revealed on TLC analysis with the solvent system of 100% toluene (Figure 1A), which enabled the separation of PQ lipids from TG, distinct from the conventional solvent system of hexane/diethyl ether/acetate [3]. Instead, the cells contained the same PQ lipid molecules as those in *Synechococystis* 6803, i.e., palmitoyl PQ-C, stearoyl PQ-C, palmitoyl PQH_2_, and stearoyl PQH_2_ on LC-MS^2^ analysis [3]. In line with this, it was deduced that *SYNPCC7002-A0918*, similar to *slr2103*, functions in the synthesis of acyl PQ-C as the PQ-C acyltransferase gene, and also in the synthesis of acyl PQH_2_. In contrast, a freshwater strain, *Synechococcus* sp. PCC 7942, possesses neither PQ lipids nor the homolog of *slr2103* [3]. However, *Synechococcus* 7942 acquires the ability to synthesize PQ lipids as novel lipids through gene manipulation to overexpress *slr2103*. In photosynthetic organisms, PQ-C is generated non-enzymatically from plastoquinone through the action of singlet oxygen that necessarily accompanies photosynthesis, whereas PQH_2_ is ubiquitous as an electron carrier [1]. Therefore, it seems probable that *SYNPCC7002-A0918* functions as a PQH_2_ acyltransferase gene as well as a PQ-C acyltransferase gene, as we previously proposed for *slr2103* [3]. The probable use of PQ-C and PQH_2_ by Slr2103 and SYNPCC7002-A0918, together with the functioning of Slr2103 as phytyl ester synthase [8], would imply their broad substrate specificity. Moreover, this study strengthened our previous idea that cyanobacterial strains, as far as those that possess homologs of *slr2103*, contain PQ lipids [3]. This idea will be evaluated in the future through PQ lipid analysis in much more cyanobacterial strains that possess *slr2103* homologs, with the use of a mass spectrometric technique that has been proven to be powerful for this purpose in this and previous studies [3]. Especially cyanobacteria such as *N. punctiforme* and *A. platensis*, which were previously reported to show TG on TLC analysis [13,14], should be examined with the possibility of considering that the reported TG represent contamination by exogenous TG and/or misidentification of PQ lipids.

PQ lipids exhibited no crucial role in cell growth under bubble aeration conditions in *Synechococcus* 7002 as well as in *Synechocystis* 6803 [3]. However, in a static culture without NaCl stress, PQ lipids contributed to the growth of sedimented cells in *Synechococcus* 7002, similar to in *Synechococystis* 6803 (Figure 5C,D) [3]. In line with this, *Synechococcus* 7002, similar to *Synechococystis* 6803, showed NaCl-stress dependent facilitation of pellicle biofilm formation in a static culture; however, the trend weakened such that the ratio of pellicle cells to total cells was 49% at most in *Synechococcus* 7002 cells at 0.3 M on Day 4 (Figure 5C,D, c.f., 76% in *Synechococystis* 6803 cells at 0.6 M on Day 15) [3]. Moreover, the responsibility of PQ lipids for pellicle biofilm formation in *Synechococcus* 7002 was not observed at 0.3 M but was observed at 0.6 M NaCl, and therefore, was restricted at severe NaCl stress relative to in *Synechococystis* 6803, which utilizes PQ lipids for acclimatization to NaCl stress at 0.3 and at 0.6 M [3]. Elucidation of the roles of PQ lipids in NaCl-stressed or non-stressed static cultures, including whether they function directly or indirectly, will be investigated in the future. In this context, however, it should be emphasized that the cellular content of PQ lipids was much lower in *Synechococcus* 7002 than in *Synechococystis* 6803 (Figure 1A,B). Habitat transition in cyanobacteria, for example, that from marine to freshwater ecoregions through evolutionary diversification, might have been accompanied by an increase in the PQ lipid content for pellicle biofilm formation and simultaneous reinforcement of NaCl-stress acclimatization ability. This thought, however, needs future evaluation through accumulation of information on other coastal and freshwater cyanobacteria.

The biosynthetic pathway for PQ-B has yet to be elucidated in seed plants. It seems reasonable to consider that PQ-C is subjected to enzymatic acylation for PQ-B synthesis in seed plants, similar to the reactions of SYNPCC7002-A0918 and Slr2103 in cyanobacteria. Future study aim to identify the gene for PQ-B synthesis in seed plants, with evaluation of phytyl ester synthase as a candidate, the amino acid sequence of which is homologous to that of Slr2103 [8], and to concomitantly explore acyl PQH_2_. Meanwhile, it is unlikely that PQ-B contributes to the physiological processes in seed plants in a similar manner to that in cyanobacteria observed under statically culturing conditions (Figure 5). It is of note that PQ-B in seed plants accepted electrons from PSII or increased in quantity during cultivation at a high light intensity [4,5], which might raise the possibility that PQ-B is involved in their acclimatization to the high-light stress. A mutant deficient in PQ lipids in *Synechococystis* 6803 or *Synechococcus* 7002 would be a powerful tool to comprehensively understand the physiological significance of PQ lipids in cyanobacteria, possibly including those in high-light stress acclimatization. In turn, such information on cyanobacteria would give a clue to find the roles of PQ-B in seed plants.

## 5. Conclusions

Euryhaline *Synechococcus* 7002 cells possess palmitoyl PQ-C, stearoyl PQ-C, palmitoyl PQH_2_, and stearoyl PQH_2_, similar to, but much less abundant, than those in freshwater *Synechocystis* 6803. An *slr2103* homolog, *SYNPCC7002-A0918*, functions as PQ-C acyltransferase for the synthesis of palmitoyl PQ-C and stearoyl PQ-C. In addition, *SYNPCC7002-A0918* also functions in the synthesis of palmitoyl PQH_2_ and stearoyl PQH_2_, probably as PQH_2_ acyltransferase. These PQ lipids play some crucial roles in sedimented cell growth in a static culture, the responsibility increasing with severe NaCl stress. However, PQ lipids at much lower levels or with less great responsibility for NaCl acclimatization in *Synechococcus* 7002 than in *Synechocystis* 6803 suggests the development of the biosynthetic system and physiological function of PQ lipids during cyanobacterial diversification. This hypothesis will be evaluated by finding PQ lipids in other cyanobacterial strains that possess orthologs of the bifunctional acyltransferase genes, and then through clarification of physiological processes that require PQ lipids.

## Figures and Tables

**Table 1:** Product ions of *m*/*z* 1007, *m*/*z* 1035, *m*/*z* 1021, *m*/*z* 1049, or *m*/*z* 766 in high-resolution MS^2^ spectra in *Synechococcus* 7002.

	16:0 PQH_2_	m/z 1007	18:0 PQH_2_	m/z 1035	16:0 PQ-C	m/z 1021	18:0 PQ-C	m/z 1049	PQ-C	m/z 766
1	m/z 153.1	+	m/z 265.3	+	m/z 151.1	+	m/z 151.1	+	m/z 151.1	+
2	m/z 237.2	+	m/z 153.1	+	m/z 109.1	+	m/z 109.1	+	m/z 95.1	+
3	m/z 109.1	+	m/z 149.1	+	m/z 95.1	+	m/z 95.1	+	m/z 191.1	+
4	m/z 149.1	+	m/z 95.1	+	m/z 149.1	+	m/z 81.1	+	m/z 109.1	+
5	m/z 95.1	+	m/z 135.1	+	m/z 81.1	+	m/z 149.1	+	m/z 149.1	+
6	m/z 123.1	+	m/z 109.1	+	m/z 135.1	+	m/z 135.1	+	m/z 135.1	+
7	m/z 81.1	+	m/z 123.1	+	m/z 123.1	+	m/z 123.1	+	m/z 123.1	+
8	m/z 135.1	+	m/z 121.1	+	m/z 121.1	+	m/z 121.1	+	m/z 81.1	+
9	m/z 163.1	+	m/z 247.2	+	m/z 163.2	+	m/z 163.1	+	m/z 121.1	+
10	m/z 151.1	−	m/z 163.1	+	m/z 203.2	+	m/z 229.1	+	m/z 163.1	+
11	m/z 137.1	+	m/z 116.1	−	m/z 189.2	+	m/z 161.1	+	m/z 177.2	+
12	m/z 177.2	+	m/z 151.1	+	m/z 137.1	+	m/z 177.2	+	m/z 217.1	+
13	m/z 121.1	+	m/z 217.2	+	m/z 191.1	+	m/z 191.1	+	m/z 189.2	+
14	m/z 191.2	+	m/z 191.2	+	m/z 177.2	+	m/z 189.2	+	m/z 161.1	−
15	m/z 219.2	+	m/z 135.1	+	m/z 161.1	+	m/z 203.2	+	m/z 175.1	+
16	m/z 203.2	+	m/z 177.2	+	m/z 175.2	+	m/z 137.1	−	m/z 147.1	−
17	m/z 189.2	+	m/z 69.1	+	m/z 147.1	−	m/z 175.1	+	m/z 203.2	+
18	m/z 217.2	+	m/z 205.2	−	m/z 107.1	+	m/z 107.1	+	m/z 107.1	+
19	m/z 175.1	−	m/z 81.1	+	m/z 229.1	+	m/z 147.1	+	m/z 116.1	−
20	m/z 97.1	+	m/z 231.2	+	m/z 191.2	−	m/z 217.2	+	m/z 137.1	+
Coverage (%)		90		90		90		95		85

## Data Availability

The data supporting the findings of this study are available from the corresponding author, N.S., upon request.
